# A new day, a new treatment. A case report

**DOI:** 10.1192/j.eurpsy.2022.1870

**Published:** 2022-09-01

**Authors:** R. Galerón, B. Serván, M. Huete Naval, E. Herrero Pellón, P. Albarracin

**Affiliations:** 1 Hospital Clínico San Carlos, Institute Of Psychiatry And Mental Health, Madrid, Spain; 2 Hospital Universitario Clínico San Carlos, Psychiatry And Mental Health, Madrid, Spain

**Keywords:** Aripiprazole, Psychosis

## Abstract

**Introduction:**

We present the case of a 21 year-old male, with history of a psychotic episode, currently with monthly follow-up in an outpatient facility, with a favorable clinical evolution after one year of intensive follow-up. In the context of abandonment of his medication and a problematic family situation, the patient starts to show suspicious, with insomnia and a progressive social isolation. Despite an attempt of ambulatory treatment with oral aripiprazole, showing good tolerance, the patient refuses such treatment, showing active clinical psychotic with great distress and behavioral repercussion, finally requiring hospital admission.

**Objectives:**

To perform a literature review about the treatment initiation with two vials of aripiprazole long-acting injection.

**Methods:**

Literature review of scientific articles using Pubmed as search engine. We considered articles published both in English and Spanish.

**Results:**

During hospital stay, treatment with 2 intramuscular injections of 400mg of aripiprazole is started, combined with a single dose of oral aripiprazole 20mg on day 1, assuring correct dosing, with good tolerance and favoring therapeutic adherence. Progressively, the patient starts to feel calmer, adequate, collaborative and emotionally stable, recuperating chronobiological rhythms, with remission of the hallucinations and appearing more distant from delusions.

**Conclusions:**

According to the currently available studies, the use of this posology could avoid the potential impact that lack of adherence to oral treatment could have in the therapeutic outcome, assuring a correct dosing and favoring adherence from day 1. Furthermore, this would help simplify the medication regiment for patients, physicians and caregivers.

**Disclosure:**

No significant relationships.

